# CXCR2: From Bench to Bedside

**DOI:** 10.3389/fimmu.2012.00263

**Published:** 2012-08-24

**Authors:** Anika Stadtmann, Alexander Zarbock

**Affiliations:** ^1^Department of Anaesthesiology, Intensive Care and Pain Medicine, University of MünsterMünster, Germany; ^2^Max-Planck Institute MünsterMünster, Germany

**Keywords:** CXCR2, chemokine receptor, Gαi-signaling

## Abstract

Leukocyte recruitment to sites of infection or tissue damage plays a crucial role for the innate immune response. Chemokine-dependent signaling in immune cells is a very important mechanism leading to integrin activation and leukocyte recruitment. CXC chemokine receptor 2 (CXCR2) is a prominent chemokine receptor on neutrophils. During the last years, several studies were performed investigating the role of CXCR2 in different diseases. Until now, many CXCR2 inhibitors are tested in animal models and clinical trials and promising results were obtained. This review gives an overview of the structure of CXCR2 and the signaling pathways that are activated following CXCR2 stimulation. We discuss in detail the role of this chemokine receptor in different disease models including acute lung injury, COPD, sepsis, and ischemia-reperfusion-injury. Furthermore, this review summarizes the results of clinical trials which used CXCR2 inhibitors.

## Introduction

Inflammation is a defense reaction caused by infection or tissue damage. The aim of this process is to eliminate the inflammatory stimulus and protect the surrounding tissue from further damage making it necessary for the survival of the host. A defect in this system can severely affect the integrity of the organism and may be fatal. This is demonstrated in patients with hereditary or acquired immune deficiency. Neutrophil depletion can have detrimental effects in some disease models, but is beneficial in others (Henson and Johnston Jr., 1987; Weiss, 1989). The reduction of neutrophil recruitment in disease models elicited by bacteria resulted in decreased bacterial clearance and reduced survival (Craig et al., 2009). The same observation can be made in patients suffering from leukocyte adhesion deficiency (LAD). This disease is characterized by a defect in leukocyte extravasation, resulting in an inappropriate inflammatory response to injury or infection (Etzioni, 2010). Patients with this disease suffer from recurrent bacterial infections and have a reduced life expectancy (Etzioni, 2010).

Neutrophil granulocytes represent an important cellular component of the innate immune system and are recruited following an inflammatory stimulus in a coordinated sequence of events into inflamed tissue (Ley et al., 2007). The leukocyte recruitment cascade consists of different steps including capturing, rolling, slow rolling, adhesion, crawling, and other activation events prior transmigration (Ley et al., 2007). Leukocyte capturing and rolling is mediated by selectins (Ley et al., 2007), whereas slow leukocyte rolling and adhesion is predominantly mediated by integrins interacting with their ligands expressed on endothelial cells. Integrins are members of a large family of conserved adhesion receptors, which occur in a low affinity conformational state on circulating leukocytes. Selectin and immunoreceptor engagement and chemokine binding to their receptors activate signaling pathways leading to the activation of integrins (inside-out signaling). During adhesion, engaged integrins can signal into leukocytes (outside-in signaling), which stabilizes adhesion and initiates transmigration. The activation of neutrophils during the recruitment process is mediated by different mediators including selectins, chemokines, and integrin-mediated outside-in signaling.

Selectin engagement activates different signaling pathways leading to tyrosine phosphorylation, cytoskeletal rearrangement, β_2_-integrin activation, cytokine secretion, and transcriptional activation. It has been shown that P- and E-selectin engagement induces β_2_-integrin activation and reduces the rolling velocity on P-selectin/E-selectin and ICAM-1 (Zarbock et al., 2007b; Kuwano et al., 2010). Selectin engagement induces LFA-1 activation in a Syk (spleen tyrosine kinase)-dependent manner (Zarbock et al., 2007b). E-selectin engagement induces the phosphorylation of the Src kinase Fgr and the ITAM (immunoreceptor tyrosine-based activation motif)-containing adaptor proteins DAP12 and FcRγ (Zarbock et al., 2008a; Yago et al., 2010). DAP12 and FcRγ subsequently recruit and activate the tyrosine kinase Syk (Zarbock et al., 2008a). In neutrophils from *Fgr^−/−^* mice and *Lyn^−/−^/Hck^−/−^* mice, DAP12, and Syk phosphorylation does not occur following E-selectin engagement (Zarbock et al., 2008a). In this signaling pathway, SLP-76 and the Tec family kinase Bruton’s tyrosine kinase (Btk) are located downstream of Syk, whereas the signaling pathway downstream of Btk divides into a phosphoinositide 3-kinase (PI3K)γ- and PLCγ2-dependent pathway (Mueller et al., 2010; Block et al., 2012). Following E-selectin engagement the small GTPase Rap1 is activated downstream of PLCγ2 (Stadtmann et al., 2011). CalDAG-GEFI (Rasgrp2) and p38 MAPK are crucial signaling molecules between PLCγ2 and Rap1a (Stadtmann et al., 2011).

During rolling, leukocytes are exposed to different chemokines and chemoattractants presented on inflamed endothelial cells. Binding of chemokines to their receptors on leukocytes activates complex intracellular signaling networks which modulate integrin activation and eventually lead to leukocyte adhesion mediated by binding of leukocyte integrins to their counter-receptors expressed on the endothelial cell surface.

Chemokine receptors are specific G protein-coupled receptors (GPCRs) on the cell surface and form specific subgroups depending on the binding capacities for members of distinct chemokine families. Chemokines are subdivided into different families depending on their structure characterized by the relative position of the first two cysteine residues of the chemokine representing the determining factor for the chemokine family classification (Baggiolini et al., 1994). For chemokines of the CC-chemokine family, the first two cysteines are adjacent to each other, whereas the first two cysteines in CXC chemokines are separated by one amino acid. Two chemokines are described so far showing a different positioning of their cysteines. Lymphotactin is characterized by the occurrence of only two cysteines and in fractalkine, the first two cysteines are separated by three amino acids (CX_3_C; Kelner et al., 1994; Bazan et al., 1997). Until now, 10 receptors for CC-chemokines (CC-chemokine receptors, CCRs), seven for CXC chemokines (CXC chemokine receptors, CXCRs), and one CX_3_C chemokine receptor (CX_3_CR) are described (Murphy, 2002; Burns et al., 2006). Chemokine receptors on the cell surface of neutrophils are exposed to different chemokines during rolling on the inflamed endothelium. Following binding of the chemokine to its receptor, intracellular signaling cascades are activated resulting in integrin activation (Zarbock et al., 2012). Neutrophils express different chemokine receptors on their surface, like CXCR1, CXCR4, CCR2, and CX_3_CR1, but for CXCR2 many different important functions are described. CXCR2 was cloned for the first time in 1991 from the human cell line HL-60 (Murphy and Tiffany, 1991). High affinity ligands for CXCR2, which is also expressed on other immune cells like mast cells, monocytes, and macrophages, are CXCL1, 2, 3, 5, 6, 7, and 8 (Olson and Ley, 2002). The most potent ligand of CXCR2 is CXCL8 as well as cleavage products of this chemokine (Van Damme et al., 1989). CXCL8 was first described and characterized as a product with chemotactic characteristics in the supernatant of LPS-stimulated human mononuclear cells in 1988 (Matsushima et al., 1988).

There is an important difference between human and murine neutrophils concerning chemokine receptor expression on the surface of neutrophils. Human neutrophils express CXCR1 and CXCR2, whereas murine neutrophils only express CXCR2, even if there are some recent reports about murine CXCR1 homologs (Fu et al., 2005; Moepps et al., 2006). High affinity ligands of CXCR1 are CXCL6 and 8 (Wolf et al., 1998). CXCL8 is the major CXCR2 ligand in humans, but in some cases, CXCL8 also binds to and mediates some functions via CXCR1. Rodents do not express CXCL8 (Reutershan, 2006).

Following adhesion, integrins may activate different signaling pathways that regulate several cellular functions including cell motility, polarization, respiratory burst, phagocytosis, proliferation, and apoptosis (Abram and Lowell, 2007). Integrin clustering and ligand-induced allosteric conformational changes likely initiate outside-in signaling and signalosome formation. The efficient protein tyrosine kinase (PTK) recruitment and activation of various signaling pathways require the formation of signalosomes (Ley et al., 2007). The two Src family kinase members Hck and Fgr are required for transducing LFA-1- and Mac-1-mediated outside-in signaling (Giagulli et al., 2006). However, these two Src family kinases are not required for chemoattractant-triggered upregulation of LFA-1 affinity and leukocyte arrest (Giagulli et al., 2006). Leukocyte adhesion strengthening can be abolished by blocking β_2_-integrin-mediated outside-in signaling (Giagulli et al., 2006). Additionally, by eliminating WASP (Sato et al., 2012), the GEFs VAV1 and VAV3 (Gakidis et al., 2004), or PI3Kγ (Smith et al., 2006) representing important signaling molecules of leukocytes, adhesion strengthening can be blocked.

## Chemokine Receptors are Characterized by Distinct Structural Properties

Chemokine receptors are normally composed of 340–370 amino acids and show a 25–80% amino acid homology (Olson and Ley, 2002). GPCRs are seven-transmembrane proteins and consist of an α-subunit and a βγ-complex whereas the classification of these receptors depends on their α-subunit. Neutrophils express G_s_-, G_i_-, and G_q_-family members with G_i_-proteins representing the most important G proteins on neutrophils. This subclass mediates almost all pro-inflammatory effects of chemoattractants and can be subdivided into different subunits (Gα_z_, Gα_o_, Gα_i1_, Gα_i2_, and Gα_i3_; Wilkie et al., 1992). With pertussis toxin (PTx), Gα_i_-signaling with the exception of Gα_z_-signaling can be blocked. Leukocytes abundantly express Gα_i2_ and Gα_i3_ (Jiang et al., 2002). A study by Zarbock et al. (2007a) demonstrated an important role for Gα_i2_ for chemokine-induced neutrophil arrest in *in vitro* and *in vivo* models. A study using Gα_i2_ deficient mice showed that Gα_i2_ in non-hematopoietic cells is involved in leukocyte migration into the lung in an allergy model and after LPS application, whereas Gα_i2_ in leukocytes is involved in regulating chemotaxis in response to chemokines (Pero et al., 2007). Gα_i3_ regulates neutrophil migration via GIV (Gα − interacting vesicle-associated protein; Ghosh et al., 2008), redistributes and localizes at the leading edge of the cell during the cell migration process (Ghosh et al., 2008). A number of Gβγ-complexes can be formed, due to the fact that leukocytes express five different β-subunits and 12 γ-subunits (Wettschureck and Offermanns, 2005).

Chemokine receptors are special GPCRs and share some structural characteristics like an NH_2_-terminal domain which is part of the chemokine binding site (Olson and Ley, 2002). They also have a conserved sequence in the second intracellular loop consisting of 10 amino acids (Olson and Ley, 2002) and share a characteristic cysteine within each extracellular domain and a short basic third intracellular loop (Murphy et al., 2000). The N-terminus of chemokine receptors which is characterized by a tyrosine sulfation motif (Murphy et al., 2000) is not important for the ligand binding affinity, however this domain is important for receptor triggering (Murphy et al., 2000). Recent studies made remarkable progress in the reconstruction of secondary and tertiary structures of GPCRs. Specialized methods like X-ray crystallography and electron microscopy revealed new insights into GPCR structures (Unger and Schertler, 1995; Pebay-Peyroula et al., 1997).

CXCR1 and CXCR2 are both Gα_i_-coupled proteins and 78% of their amino acid sequences are identical (Reutershan, 2006). Divergent regions carrying the differences between the two receptors are the N-terminus, the C-terminus, the second extracellular loop, and the fourth transmembrane domain (Nasser et al., 2007). These regions are probably responsible for functional differences between CXCR1 and CXCR2 which will be mentioned later on. CXCR2 is a member of the rhodopsin-like family of GPCRs, however the crystal structure of CXCR2 is not revealed, yet (Murphy et al., 2000).

## GPCR-Induced signaling

Binding of a chemokine to its receptor may induce many different cellular responses like adhesion, migration, and chemotaxis including cellular shape changes, reorganization of the actin cytoskeleton, upregulation of integrin expression, and integrin activation (Baggiolini, 1998).

**Figure 1:**
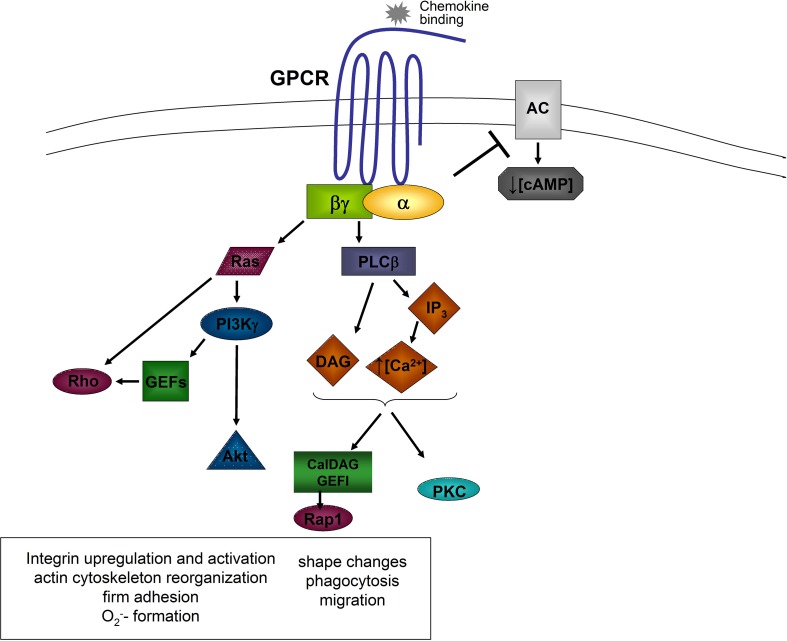
**General model of chemokine-induced signaling**. Downstream of the α and βγ subunits, different signaling molecules are activated leading to different cellular functions. AC, adenylyl cyclase; Akt, proteine kinase B; Ca^2+^, calcium; cAMP, cyclic adenine monophosphate; DAG, diacylglycerol; GEFs, guanine exchange factors; GPCR, G protein coupled receptor; IP_3_, inositol triphosphate; PI3K, phosphatidylinositol-3-kinase; PKC, protein kinase C; PLCβ, phospholipase C beta; Rho, Ras, Rap1, small G proteins.

The activation of a GPCR by the engagement of a chemoattractant results in an activation of the associated G protein, which dissociates into the GTP-bound Gα-subunit and the Gβγ-complex (Zarbock and Ley, 2008). Both, the α-subunit and the βγ-complex, are able to activate different signaling molecules (Figure 1).

Following dissociation, the α-subunit inhibits some adenylyl cyclase isoforms leading to a decrease of intracellular cAMP-levels and cAMP-dependent protein kinase activity (Sunahara et al., 1996). Gα_i_-subunits are also involved in the activation of small GTPases following GPCR activation. Gα_i_-dependent Ras activation induces the activation of phosphatidylinositol-3-kinase (PI3K) by directly binding to the catalytic subunit of PI3K (Rodriguez-Viciana et al., 1994).

The βγ-complex is able to activate PI3Kγ and the two phospholipase C (PLC) isoforms β_2_ and β_3_ (Camps et al., 1992; Hirsch et al., 2000). PLC hydrolyzes phosphatidylinositol 4,5-bisphosphate to generate inositol trisphosphate (IP_3_), which mobilizes calcium from non-mitochondrial stores, and diacylglycerol (DAG), which activates Ca^2+^-independent and Ca^2+^-dependent protein kinase C (PKC; Berridge and Irvine, 1984). Different PKC isoenzymes are required for activating cytotoxic effector functions of neutrophils (Mayer et al., 1996). PLC β_2_-deficient neutrophils show enhanced chemotaxis and increased leukocyte recruitment in response to fMet-Leu-Phe (fMLP), but reduced chemoattractant-induced Ca^2+^ release and macrophage-1 antigen (Mac-1) upregulation (Jiang et al., 1997). Murine neutrophils deficient in both, PLC β_2_ and PLC β_3,_ show impaired chemokine-stimulated O_2_^−^ formation (Li et al., 2000). It has also been shown that PLC is involved in chemokine-induced α_4_β_1_-integrin activation and monocyte adhesion (Hyduk et al., 2007). A recent study by Stadtmann et al. (2011) demonstrated that the GEF CalDAG-GEFI, which requires Ca^2+^ and DAG for activation, is necessary for chemokine-induced neutrophil arrest *in vivo*.

PI3Kγ, which can be activated by different βγ-subunits, catalyzes the phosphorylation of phosphatidylinositol-3,4-bisphosphate (PIP_2_) to phosphatidylinositol-3,4,5-trisphosphate (PIP_3_) which is able to bind to proteins containing pleckstrin homology domains, leading to downstream signaling (Varnai et al., 1999). Such an example for PI(3)Kγ-dependent downstream signaling is the activation of Akt, which clusters at the leading edge of migrating neutrophils (Figure 1; Servant et al., 2000).

The small GTPases of the Rho-family are also activated downstream of PI3Kγ (Servant et al., 2000). Neutrophils express Rac1 and Rac2 and both molecules regulate the migration of neutrophils, whereas Rac2 is also involved in the regulation of the respiratory burst (Glogauer et al., 2003; Gu et al., 2003). The activation of Rac is mediated by guanine nucleotide exchange factors (GEFs) by exchanging GDP for GTP. Different GEFs are expressed in neutrophils and these molecules are involved in Rac activation like the GEFs of the P-Rex family which are directly activated by the Gβγ subunit and PIP_3_. In contrast, Vav family GEFs in neutrophils are activated in a Syk and Src kinases dependent pathway (Figure 1; Welch et al., 2002; Fumagalli et al., 2007). A recent study by Lawson et al. (2011) demonstrated that P-Rex-1- and Vav-1-deficiency mediates severe defects in GPCR dependent neutrophil activation. Another important molecule called DOCK2 (dedicator of cytokinesis 2), can also regulate the activity of Rac1 and Rac2 (Kunisaki et al., 2006) and influences cell polarity changes and translocation speed (Kunisaki et al., 2006). Activated Rac activates p21-activated kinase (PAK), which can subsequently induce the phosphorylation of extracellular signal-regulated kinase 1/2 (ERK1/2), p38 mitogen-activated protein kinase, and c-Jun N-terminal kinase (Kim and Dinauer, 2001; Fumagalli et al., 2007).

CXCR2-triggered signaling uses the same molecules described above, but a recent study by Wu et al. (2012) revealed a new mechanism for the coupling of CXCR2 to its downstream signaling molecules. They showed that the PDZ scaffold protein Na^+^/H^+^ exchanger regulatory factor-1 (NHERF1) couples CXCR2 to its downstream effector PLC-β2, forming a macromolecular complex, through a PDZ-based interaction (Wu et al., 2012). Disruption of this complex led to a decrease of intracellular calcium concentrations on the molecular level, and suppressed neutrophil chemotaxis and migration on the cellular level following chemokine stimulation (Wu et al., 2012).

There are differences described for the signaling cascades downstream of either CXCR1 or CXCR2 activation. In contrast to CXCR2 dependent signaling, phospholipase D gets activated and neutrophils are primed to perform respiratory burst following CXCR1 activation (L’Heureux et al., 1995; Jones et al., 1996). Due to these data, it is likely that the activation of the two receptors play different roles under inflammatory conditions.

## CXCR2 is Involved in Physiological and Pathological Conditions

Neutrophils are essential for maintaining innate immune surveillance under normal conditions, but also represent a major contributor to tissue damage during autoimmune processes. Therefore, neutrophil homeostasis and recruitment are tightly regulated. CXCR2 plays a critical role in the regulation of neutrophil homeostasis (Eash et al., 2010; von Vietinghoff et al., 2010; Mei et al., 2012), because CXCR2-deficient mice demonstrate mild neutrophilia and severe neutrophil hyperplasia in the bone marrow (Shuster et al., 1995). A recent study demonstrated that CXCR2-signaling is a second chemokine axis that interacts antagonistically with CXCR4 to regulate neutrophil release from the bone marrow (Eash et al., 2010).

During inflammation, leukocyte extravasation from the blood vessel into inflamed tissue is one of the hallmarks of the immune system. However, leukocyte recruitment has to be tightly regulated, as excessive leukocyte extravasation may lead to the deterioration of the integrity of the organism and may worsen acute and chronic inflammatory diseases. Different chemokines, which are released during inflammation, direct leukocytes to the site of inflammation. The chemokine receptor CXCR2 and its ligands have been implicated in a variety of inflammatory disorders making it an interesting target for therapeutical approaches (Seitz et al., 1991; Boyle Jr. et al., 1998; Kurdowska et al., 2001; Bizzarri et al., 2006; Reutershan, 2006; Chapman et al., 2009). In several inflammatory disease models, blocking, or eliminating CXCR2 substantially reduces leukocyte recruitment, tissue damage, and mortality. Based on the physiological importance of CXCR2, selective CXCR2 inhibitors have been developed that are now being tested in clinical trials. Due to the structural similarities between CXCR1 and CXCR2 or an influence by the activity of one of these receptors on the activity of the other, CXCR2 manipulation may affect CXCR1 dependent functions. This possibility has to be tightly controlled, but experiments in the murine system do not include these cross-reactivities because murine neutrophils do not express CXCR1. The following part summarizes current knowledge about CXCR2 in inflammatory diseases and discusses its potential as a pharmaceutical target.

### Lung diseases

CXCR2 is found on many cells including leukocytes, endothelial, and epithelial cells. On endothelial cells, CXCR2 expression was demonstrated for the human and murine system (Murdoch et al., 1999; Addison et al., 2000). Additionally it was demonstrated that CXCR2 expression is important for angiogenesis and supports tumor growth (Addison et al., 2000; Keane et al., 2004). Lung epithelium of COPD patients expresses elevated levels of CXCR2, but not CXCR1, indicating different roles for these two receptors under COPD disease conditions (de Boer, 2002; Qiu et al., 2003).

Due to its expression on lung endothelial and epithelial cells, it is not surprising that CXCR2 has been implicated in different lung diseases. Several studies have identified an important role for CXCR2 in acute lung injury (ALI), asthma, COPD, and cystic fibrosis (CF).

### Acute lung injury

Acute lung injury is characterized by a damage of the alveolar-capillary barrier resulting in infiltration of neutrophils into the lungs, pulmonary edema, remodeling of the alveolar and small airway epithelium, and collagen deposition in the pulmonary interstitium. Lung fibrosis can result from ALI (Ley and Zarbock, 2008; Chapman et al., 2009). ALI may completely resolve or proceed to fibrosing alveolitis accompanied by persistent low oxygen in the blood (hypoxemia) and a reduced ability of the lung to expand with every breath (reduced pulmonary compliance; Rubenfeld et al., 2005). In both pulmonary diseases, a functional role for CXCR2 has been implicated.

Acute lung injury is a common disease with an incidence of 79 per 100,000 person-years in the United States (Rubenfeld et al., 2005). Despite the use of state-of-the-art treatment, this disease is associated with high mortality of up to 38% (Rubenfeld et al., 2005). Pneumonia and acid aspiration are intrapulmonary causes of ALI whereas trauma, massive transfusion, and sepsis are typical causes of extrapulmonary ALI. During ALI, neutrophils are recruited in the alveolar compartment and increased CXCL8 levels in the BAL fluid of patients with ALI have been positively correlated with the presence of activated neutrophils (Aggarwal et al., 2000; Kurdowska et al., 2001; Keane et al., 2002; Puneet et al., 2005). High levels of CXCL8 complexed with anti-CXCL8 autoantibodies were found in the alveolar fluid of patients suffering from ALI (Fudala et al., 2007). As these complexes can inhibit neutrophil apoptosis (Fudala et al., 2007), it is possible that this condition may prolong neutrophil survival and exacerbate the deleterious effects of neutrophil activation. These data identify CXCL8 as an important chemokine in the pathogenesis of ALI (Puneet et al., 2005). In addition to this, CXCL8 has also been associated with other pathophysiological aspects of ALI. Due to an increase in vascular permeability, higher levels of α_2_ macroglobulin, and CXCL2 are found in the BAL of of patients with ALI (Kurdowska et al., 1997). CXCL8 and α_2_ macroglobulin form complexes which maintain chemoattractant activity (Kurdowska et al., 2001). Based on this fact, it is likely that the complex perpetuates the inflammatory response in the lungs of ALI patients. Patients with CXCL8 gene polymorphisms accompanied with higher levels of CXCL8 have more prolonged and extensive lung injury which requires an extended time on ventilatory support (Hildebrand et al., 2007).

In animal models of ALI, a very important role for CXCL8 and CXCR2 has been clearly identified. Exposure to hyperoxic gas (Sue et al., 2004) and ventilator-induced lung injury (Strieter et al., 2005b) caused neutrophil recruitment into the lung, increased airway microvascular leakage, and induced lung edema. Each of these pathological changes was diminished in WT animals treated with neutralizing antibodies to CXCR2 or in CXCR2-deficient mice. Similar findings were observed following blocking CXCR2 in animal models of lung injury induced by lipopolysaccharide (LPS; Chapman et al., 2007), after lung infection with viral or bacterial challenge (Tsai et al., 2000; Del et al., 2001; Londhe et al., 2005b; Strieter et al., 2005a) and after induction of hemorrhagic shock (Lomas-Neira et al., 2004).

Angiogenesis is considered to be an important component of collagen deposition and fibrosis formation in the lungs (Keane et al., 2004). A variety of CXC chemokines possessing angiogenic properties are expressed in pulmonary edema fluid of patients with ALI (Keane et al., 2002) with CXCR2 as an important receptor mediating this effect (Addison et al., 2000). These data suggest that inhibition of CXCR2 may also be beneficial in reducing the development of pulmonary fibrosis in ALI patients.

### Chronic obstructive pulmonary disease

Chronic obstructive pulmonary disease (COPD) is characterized by a limitation of the airflow that is not fully reversible, and is usually progressive with an abnormal inflammatory response (Rabe et al., 2007). This disease is primarily caused by cigarette smoking where recurrent lung infections may induce a progressive decline in lung function. Small airway disease (obstructive bronchiolitis) and parenchymal destruction (emphysema) are the causes of the limited airflow. Bacterial and viral infections are frequent causes of COPD exacerbations. No medication exists that prevents the long-term decline in lung function, however at the present time, inhaled anticholinergics, β-adrenergic bronchodilators, and corticosteroids are used to treat the symptoms and exacerbations of COPD.

Chronic bronchitis is associated with excess mucus secretions in the large airways and a large number of recruited leukocytes, especially neutrophils (de Boer et al., 2000; Rabe et al., 2007). As chronic bronchitis is associated with mucus hypersecretion and neutrophil recruitment, it is not surprising that CXC chemokines and CXCR2 expression in the bronchial biopsies and sputum of COPD patients are increased (Traves et al., 2002; Qiu et al., 2003). Animal studies using lung infection models demonstrated that CXCR2 has an important role in this response (Tsai et al., 2000; Del et al., 2001; Miller et al., 2003; Londhe et al., 2005a). Similar observations can be made in humans suffering from COPD. Increased CXCR2 mRNA expression is present in bronchial biopsy specimens from COPD patients which goes along with the presence of tissue neutrophils during severe exacerbations of COPD (Qiu et al., 2003). These data suggest that CXCR2 inhibition may be a viable therapeutic approach against the inflammatory events occurring in the distal lungs of human COPD patients.

In contrast to the mucus hypersecretion in the large conducting airways, the presence of inflammatory mucus exudates in the peripheral airways contributes significantly to the airflow obstruction in COPD (Hogg et al., 2004). In animal studies using different COPD models, blocking CXCR2 inhibited mucus hypersecretion, and goblet cell hyperplasia (Miller et al., 2003; Stevenson et al., 2005; Chapman et al., 2007). However, these animal models investigated the mucus production in the large conducting airways, where the mucus rarely leads to airflow obstruction. Therefore, it will be important to assess the effect of CXCR2 blockade on mucus inflammatory exudates and obstruction in the peripheral airways.

### Asthma

Asthma is a common chronic inflammatory disease of the airways characterized by variable and recurring symptoms, reversible airflow obstruction, bronchial hyperresponsiveness, chronic eosinophilic lung inflammation, and bronchospasm (Bousquet et al., 2000). Symptoms include coughing, wheezing, chest tightness, and shortness of breath (Bousquet et al., 2000). Mild to moderate asthma is treated with a combination of inhaled beta-adrenergic bronchodilators and corticosteroids. However, severe asthmatics only respond poorly to inhaled beta-adrenergic agonists and corticosteroids. Severe asthma is characterized by a predominantly neutrophilic inflammation of the lung with airway remodeling.

During severe asthma, a positive correlation between increased expression of ELR^+^ CXC chemokines, which carry the Glu-Leu-Arg (ELR) tripeptide motif at the NH2-terminus, and the presence of neutrophils in the lung exists (Kurashima et al., 1996; Lamblin et al., 1998; Norzila et al., 2000; Pease and Sabroe, 2002; Mukaida, 2003). In addition, it has been shown that increases in sputum CXCL8 precede the exacerbations of acute asthma (Mukaida, 2003). Viral and bacterial lung infections contribute significantly to the frequency of asthma exacerbations and studies in animals have shown an important role for CXCR2 in this response.

Eosinophils are present in sputum and bronchial biopsies of patients with mild to moderate asthma. Under physiological conditions, the expression and role of CXCR2 on eosinophils is uncertain. However, it is possible that this receptor plays a role on these cells under conditions of chronic lung inflammation. Given the fact that CXCR2 is highly expressed on the vascular endothelium and that animal studies have demonstrated a role for endothelial CXCR2 on mast cell migration into tissue following sensitization to allergen (Hillyer et al., 2003; Abonia et al., 2005; Hallgren et al., 2007), this may be important in inducing acute phase responses to allergen challenge.

The increased formation of blood vessels in airway mucosa with associated changes in the vascularity is a characteristic of human asthma (McDonald, 2001; Hashimoto et al., 2005). Th_2_-dependent cytokines induce the synthesis of angiogenic chemokines (Matsuda et al., 2008), and different studies demonstrated that CXCR2 and its ligands are involved in the formation of new blood vessels in the lungs (Addison et al., 2000; Belperio et al., 2000; Babu et al., 2007; Mohsenin et al., 2007). CXCR2 is also expressed on airway smooth muscles and it could be speculated that the receptor is involved in the contractile and migratory responses of airway smooth muscle in chronic asthma (Govindaraju et al., 2006). This also raises the possibility that CXCR2 on airway smooth muscle cells is involved in the development of bronchial hyperresponsiveness in asthmatics. Indeed, inhalation of CXCL8 causes bronchoconstriction in pigs (Fujimura et al., 1999).

These data identify an important role for the CXC chemokines and CXCR2 in lung inflammation, lung histopathology, and abnormal physiology that is seen in asthma.

### Cystic fibrosis

Cystic fibrosis is an autosomal genetic disease affecting most critically the lungs, and also the pancreas, intestine, and liver. CF is caused by a mutation in the gene for the protein CF transmembrane conductance regulator (CFTR) and the disease is characterized by abnormal transport of chloride and sodium across epithelium, leading to thick, viscous secretions (Clunes and Boucher, 2007). The absence or lack of functional CFTR in the airway epithelium leads to dysfunctional lung mucociliary clearance, recurrent lung infections, hypertrophy and hyperplasia of mucus secreting cells and glands, and small airway obstruction. The airways of patients suffering from CF are frequently infected with bacterial pathogens, which determine morbidity and mortality in these patients (Chapman et al., 2009). As a consequence of the airway infection, pro-inflammatory cytokines, and chemokines, including CXCL8, are produced that attract large numbers of neutrophils into the lung (Dean et al., 1993; Elizur et al., 2008). However, it is very likely that a variety of chemokines are involved in the pathology of CF as several non-ELR^+^ CXC chemokines are also known to play a role in the inflammatory process of CF (Mackerness et al., 2008).

In CF patients, high levels of neutrophil elastase are found in airway secretions (Goldstein and Doring, 1986) which probably participates in the elevated mucus secretion in these patients (Voynow et al., 2004; Tirouvanziam et al., 2008). Studies of different animal models indicated that CXCR2 is involved in mucus hypersecretion and proliferation of mucus secreting cells in the airway. Neutrophils are also important for the antimicrobial response of the lungs (Tsai et al., 2000; Hartl et al., 2007). However, recently published studies suggest that CXCR1 rather than CXCR2 is the functionally important receptor involved in neutrophil degranulation (Geiser et al., 1993; Jones et al., 1996; Patel et al., 2001; Feniger-Barish et al., 2003). Therefore, blocking CXCR2 should not affect neutrophil phagocytosis and mediator release. As there is evidence showing the cleavage of CXCR1 on neutrophils, which disables the bacterial-killing capacity of neutrophils from CF patients (Hartl et al., 2007), the incidence of infection should be closely monitored.

### Sepsis

Sepsis is a major healthcare problem, affecting millions of individuals around the world each year, killing one in four, and increasing in incidence (Dombrovskiy et al., 2007; Andaluz-Ojeda et al., 2011). The immune system combats microbial infections but, in severe sepsis, its untoward activity seems to contribute to organ dysfunction. The inappropriate activation and positioning of neutrophils within the microvasculature contributes to the pathological manifestations of multiple organ failure.

Cummings et al. (1999) showed that the expression levels of CXCR2 on circulating neutrophils of septic patients are decreased by approximately 50% in comparison to control donors. This was associated with a reduced migratory activity of neutrophils toward ligands specifically binding to CXCR2 (CXCL1-3 and CXCL5), while migration toward IL-8 was unaffected (Cummings et al., 1999). The expression level of CXCR1 did not show any significant alterations, suggesting an important role of CXCR1 in septic patients (Cummings et al., 1999). CXCR2 downregulation can be explained by the high levels of soluble chemokines circulating within the plasma of septic patients (Phillipson and Kubes, 2011).

While CXCR2 is important for leukocyte extravasation into inflamed tissue and might be highly relevant for bacterial clearance and survival in bacteria-induced pulmonary inflammation, it has deleterious effects in sepsis. Blocking or eliminating CXCR2 decreased liver injury and mortality in a murine model of cecal ligation and puncture (CLP)-induced sepsis (Ness et al., 2003). Although neutrophil recruitment into the peritoneum was delayed, bacterial clearance was not affected by eliminating CXCR2. Application of cell-penetrating lipopeptides, which block CXCR1- and CXCR2-signaling, reversed the lethal sequelae of sepsis, including multi-organ failure and disseminated intravascular coagulation in mice, indicating that CXCR2 is very important in sepsis (Kaneider et al., 2005).

### Reperfusion-injury

Ischemia-reperfusion-injury contributes to morbidity and mortality in a wide range of pathologies, including circulatory arrest, ischemic stroke, myocardial infarction, acute kidney injury, and trauma. Additionally, it is a common challenge during cardiothoracic and vascular surgery and organ transplantation. Due to the reduced metabolic supply in the ischemic organ, tissue hypoxia, and microvascular dysfunction occur. The subsequent reperfusion activates an innate and adaptive immune response (Eltzschig and Eckle, 2011) with a characteristic strong accumulation of inflammatory cells, predominantly neutrophils, into the injured organs leading to tissue injury (Eltzschig and Eckle, 2011).

Since neutrophil infiltration is a major cause of tissue injury, mechanisms involved in neutrophil recruitment following an inflammatory stimulus are interesting targets for therapeutic approaches. Several recent studies demonstrated a positive effect of inhibiting neutrophil recruitment into the region of ischemia-reperfusion (Kempf et al., 2011; Block et al., 2012).

Neutrophil depletion reduces tissue injury after myocardial ischemia-reperfusion in patients (Palatianos et al., 2004) and animals (Litt et al., 1989). In a model of myocardial infarction, Tarzami et al. (2003) demonstrated that the infarct size in CXCR2-deficient mice is significantly reduced in comparison to wildtype mice, predominantly mediated by CXCR2 on hematopoietic cells. More precise, investigation of the infarcted zone revealed a decreased number of infiltrated immune cells in CXCR2^−/−^ mice (Tarzami et al., 2003). This finding is consistent with the important role of CXCR2 in leukocyte recruitment into inflamed tissue.

During organ transplantation, reperfusion-injury mediated by neutrophils is a major challenge, because it is associated with increased morbidity and mortality (King et al., 2000). Neutrophil accumulation in the kidney occurs rapidly after reperfusion, is associated with an increased CXCR2 and CXCL1 expression in the graft and is an important predictor of delayed graft function after kidney transplantation (Turunen et al., 2004). Blocking CXCR2 by an inhibitor reduces neutrophil accumulation in the kidney and maintains kidney function (Cugini et al., 2005). Inhibition of CXCR2 also reduced neutrophil recruitment and organ dysfunction in other models of ischemia-reperfusion-injury (Bertini et al., 2004; Souza et al., 2004; Belperio et al., 2005).

## Possible Therapeutic Strategies by Blocking CXCR2

As described above, CXCR2 and CXCR2 ligands are involved in many processes which can influence different disease conditions. Therefore, controlling CXCR2 and therewith CXCR2 dependent processes can be powerful therapeutic mechanisms. Affecting CXCR2 dependent pathways is possible in different ways. Distinct strategies including N-terminally modified chemokines, antibodies, and small-molecule antagonists were tested.

Several low molecular weight CXCR2 antagonists have been developed and tested in different *in vitro* and *in vivo* models. The first low molecular weight CXCR2 antagonist was described in 1998 in a study by White et al. (1998). The described antagonist was a selective non-peptide antagonist of CXCR2 and inhibited CXCL8 and GROα dependent neutrophil chemotaxis *in vitro*. Therefore, it was suggested as a potential tool for therapeutic application (White et al., 1998). Following the identification of this compound, a class of diarylureas was tested in different disease models for possible therapeutic usage.

Another type of CXCR2 inhibitors are allosteric inhibitors, which block CXCR2 function by blocking receptor signaling instead of chemokine binding. In the beginning of allosteric inhibitor investigation, a concentration-dependent inhibitory effect on CXCL8 function, such as neutrophil chemotaxis was reported (Souza et al., 2004). Non-steroidal anti-inflammatory drugs known as ketoprofen and ibuprofen were reported to be potent inhibitors of CXCL8 dependent neutrophil chemotaxis (Bizzarri et al., 2001). Subsequently, a series of potent allosteric CXCR2 inhibitors were described. Another non-competitive allosteric inhibitor is Reparixin (or Repertaxin), which specifically blocks CXCR1 or CXCR2-mediated neutrophil migration *in vitro* without affecting other receptors (Bertini et al., 2004). It was previously demonstrated that Reparixin inhibits CXCL8 induced neutrophil activation and blocks the increase of intracellular free calcium, elastase release, and production of reactive oxygen intermediates (Bertini et al., 2004). The application of this inhibitor in different disease models demonstrated that Reparixin is able to mediate beneficial effects in a bacteria-induced peritonitis, a venom-induced lung injury, and different models of ischemia-reperfusion-injury (Bertini et al., 2004; Souza et al., 2004; Cugini et al., 2005; Garau et al., 2005; Coelho et al., 2007). A recently published study by Zarbock et al. (2008b) demonstrated that Reparixin attenuates ALI by reducing neutrophil recruitment and vascular permeability. Because of suboptimal pharmacokinetic characteristics of Reparixin, related compounds were tested and DF 2162 was reported to have similar effects as Reparixin, but better pharmacokinetic characteristics (Coelho et al., 2007; Cunha et al., 2008; Chapman et al., 2009).

The pyrimidine series-based CXCR2 antagonist AZD-8309 has been clinically tested in different disease models. A recent report by Virtala et al. (2011) reported that AZD-8309 application reduced LPS-induced neutrophil recruitment and elastase activity about 50% in comparison to placebo application in a lower airway LPS model.

Different CXCR2 antagonists developed by GlaxoSmithKline were also reported to be able to inhibit chemokine binding to CXCR2, mediating decreased migration in response to CXCL1, 5, and 7 (Traves et al., 2004). Another antagonist was demonstrated to inhibit CD11b upregulation and shape changes as characteristics of neutrophil activation following stimulation with CXCL1 of neutrophils from COPD patients (Chapman et al., 2009). One of the antagonists of this group, SB-656933, was already tested in clinical trials of CF and ozone-induced tissue injury. In this study, oral administration of SB-656933 inhibited CXCL1-induced CD11b upregulation on neutrophils and reduced ozone-induced airway inflammation in a dose-dependent manner. This was quantified by neutrophil counts in the sputum following ozone challenge (Lazaar et al., 2011).

Another recent study reported that the CXCR2 antagonist SCH527123, which is a potent inhibitor of neutrophil activation and trafficking in animal models, is a potent inhibitor of ozone-induced neutrophil recruitment in a human clinical trial (Holz et al., 2010). SCH527123 treatment of healthy humans resulted in significantly lower sputum total cell and neutrophil counts as well as CXCL8 levels following ozone treatment in comparison to prednisolon or placebo treated study participants (Holz et al., 2010).

GSK1325756, a specifically designed CXCR2 antagonist, was already tested in a phase I clinical trial, determining the effects of GSK1325756 in healthy adult volunteers (www.clinicaltrials.gov, study number NCT01267006). In this study, blood parameters were checked in participants treated with either a placebo or GSK1325756. In another group the effect of food to the levels and outcome of GSK1325756 was investigated.

As CXCR2 is involved in the pathogenesis of many diseases, this receptor and its ligands are interesting targets for clinical trials. However, it has to be kept in mind that blocking CXCR2 can have beneficial but also harmful effects. Blocking CXCR2 inhibits the inflammatory response. Following bacterial or viral infections, the inhibition of the immune response can be very dangerous. Pathogens cannot be eliminated accurately, leading to dissemination of the pathogen and systemic infection. On the other hand, under sterile inflammatory conditions, for example following ischemia-reperfusion-injury, decreasing the neutrophil recruitment and the immune response can be beneficial. Further studies and clinical trials are necessary to further elucidate the exact effects of blocking CXCR2 and/or its ligands under different disease conditions.

## Conflict of Interest Statement

The authors declare that the research was conducted in the absence of any commercial or financial relationships that could be construed as a potential conflict of interest.
